# Association of TyG and TyG/HDL with *Helicobacter pylori* infection status and urea breath test load: a cross-sectional study

**DOI:** 10.3389/fmed.2026.1824476

**Published:** 2026-05-08

**Authors:** Yu Zhou, Ge Yu, Rong Wan

**Affiliations:** 1 Shanghai General Hospital of Nanjing Medical University, Shanghai, China; 2 Department of Gastroenterology, Xuancheng People’s Hospital, Affiliated Xuancheng Hospital Wannan Medical College, Xuancheng, Anhui, China; 3 Department of Gastroenterology, Shanghai General Hospital, Shanghai Jiao Tong University School of Medicine, Shanghai, China

**Keywords:** 13C-urea breath test, dose–response relationship, *Helicobacter pylori*, insulin resistance, triglyceride–glucose index, TyG/HDL-C ratio

## Abstract

**Objective:**

To evaluate the associations of the triglyceride–glucose index (TyG) and the TyG/high-density lipoprotein cholesterol ratio (TyG/HDL-C) with Helicobacter pylori infection status and the corresponding dose–response pattern, and to explore whether bacterial activity–related load, as reflected by delta over baseline (DOB) is associated with TyG and TyG/HDL-C among *H. pylori*–positive individuals.

**Methods:**

We retrospectively screened 705 hospitalized patients at our center. *H. pylori* positivity was defined as a delta over baseline (DOB) ≥ 4‰ on the ^13^C-urea breath test (^13^C-UBT). Logistic regression was used to estimate odds ratios (ORs) for *H. pylori* positivity in relation to TyG and TyG/HDL-C, and restricted cubic splines (RCS) were applied to assess potential nonlinearity. Among *H. pylori*–positive participants, DOB was used as a proxy for bacterial activity–related load. We examined associations of ln(DOB) with TyG and TyG/HDL-C using linear regression, conducted sensitivity analyses, and performed additional subgroup and interaction analyses stratified by age and BMI.

**Results:**

Of 705 participants, 238 were *H. pylori*–positive (33.8%). TyG was independently associated with *H. pylori* positivity (OR 1.472, 95% CI 1.096–1.978; *P* = 0.010), and RCS suggested a nonlinear association (P for nonlinearity = 0.005). TyG/HDL-C was associated with *H. pylori* positivity in unadjusted and partially adjusted models (OR 1.239; *P* = 0.011) but was not significant after full adjustment (OR 1.158; *P* = 0.102). Additional subgroup analyses showed that the association between TyG and *H. pylori* positivity was stronger among participants with BMI ≥ 24 kg/m^2^ (P for interaction = 0.009), whereas the association between TyG/HDL-C and *H. pylori* positivity varied by age (P for interaction = 0.025). Among *H. pylori*–positive participants, ln(DOB) was not significantly associated with TyG or TyG/HDL-C, and results were unchanged after excluding extreme DOB values. The association between TyG and *H. pylori* positivity remained robust after excluding participants with diabetes and those with extreme triglyceride values.

**Conclusion:**

TyG is independently associated with H. pylori infection status with a nonlinear dose–response pattern. The association between TyG/HDL-C and *H. pylori* positivity was not significant after full adjustment. No clear association is observed between DOB and TyG or TyG/HDL-C among *H. pylori*–positive individuals.

## 1 Introduction

*Helicobacter pylori* is a Gram-negative bacterium that primarily colonizes the stomach, infecting approximately half of the global population (1). *H. pylori* infection can trigger gastric mucosal inflammation and is closely associated with the development and progression of gastritis, peptic ulcer disease, and gastric cancer (2). The triglyceride–glucose (TyG) index, calculated from fasting triglycerides and glucose, is a simple surrogate marker of insulin resistance (3), and the TyG/HDL-C ratio may provide additional value for cardiometabolic risk stratification (4). In recent years, accumulating evidence has suggested that *H. pylori* infection is associated with metabolic abnormalities and insulin resistance (5). However, prior studies have reported inconsistent findings regarding the relationship between *H. pylori* infection and metabolic status (5), and few investigations have examined these associations from the perspective of DOB. Therefore, this study aimed to evaluate the relationships of TyG and TyG/HDL with *H. pylori* infection status, characterize the dose–response pattern, and further explore whether DOB is associated with TyG and TyG/HDL.

## 2 Materials and methods

### 2.1 Study design and participants

**FIGURE 1:**
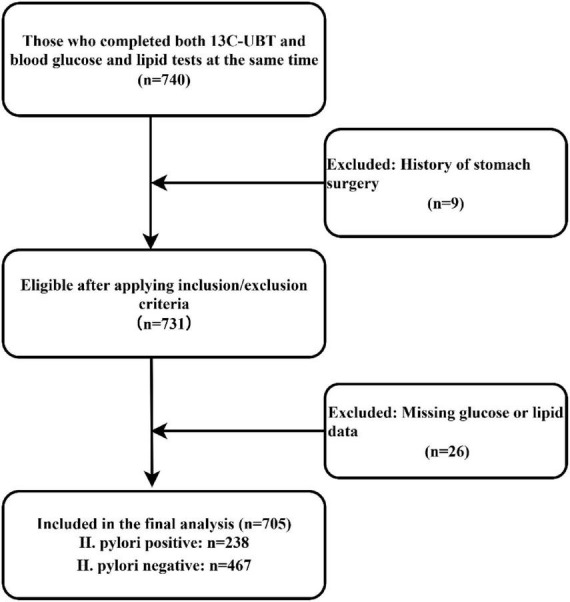
Inclusion and exclusion flowchart.

This was a retrospective cross-sectional study. We retrospectively identified hospitalized patients at our center who underwent the ^13^C-urea breath test and concurrent blood glucose and lipid testing during the same hospitalization. A total of 740 patients were screened. Nine patients with a history of gastric surgery were excluded. Twenty-six patients were excluded due to missing key glucose and/or lipid data. Ultimately, 705 patients were included in the final analysis. The study flow is shown in Figure 1.

### 2.2 Data collection and definitions

#### 2.2.1 Clinical and laboratory data

Demographic and clinical characteristics, including sex, age, height, weight, and histories of smoking, alcohol use, hypertension, and diabetes (coded as binary variables), were extracted from the electronic medical record system. Laboratory measurements, including blood glucose and lipid profiles, were collected concurrently.

#### 2.2.2 *Helicobacter pylori* assessment

All participants underwent ^13^C-UBT. According to the routine clinical testing protocol, recent use of PPIs, bismuth compounds, and antibiotics was avoided before testing. The test was performed as follows. Participants fasted for at least 2 h, after which a baseline breath sample was collected at rest. After ingestion of 75 mg of ^13^C-urea, a second breath sample was collected 30 min later. Samples were analyzed by mass spectrometry, and delta over baseline (DOB) values were calculated. *H. pylori* positivity was defined as DOB ≥ 4‰.

#### 2.2.3 Biochemical measurements and index calculations

Fasting venous blood samples were collected in the morning. Serum lipid levels were measured using enzymatic assays. The TyG index was calculated as: TyG = ln [TG (mg/dL) × glucose (mg/dL)/2]. The TyG/HDL-C ratio was defined as TyG/HDL-C = TyG / HDL-C (mg/dL).

### 2.3 Statistical analysis

#### 2.3.1 Descriptive statistics

Statistical analyses were performed using SPSS (version 25.0) and Python (version 3.12). Continuous variables are presented as mean ± standard deviation or median (interquartile range), as appropriate. Categorical variables are presented as number (percentage). Baseline characteristics were compared between *H. pylori*–negative and *H. pylori*–positive groups. Continuous variables were compared using the *t*-test or Mann–Whitney U test, and categorical variables were compared using the χ^2^-test. All tests were two-sided, and *P* < 0.05 was considered statistically significant.

#### 2.3.2 Associations of TyG and TyG/HDL-C with *H. pylori* positivity

*H. pylori* positivity was modeled as the outcome using binary logistic regression, with TyG and TyG/HDL-C as exposures. Odds ratios (ORs) with 95% confidence intervals (CIs) were reported. Three models were fitted to control for potential confounding: Model 1, unadjusted; Model 2, adjusted for age and sex; and Model 3, further adjusted for BMI, smoking, alcohol use, hypertension, and diabetes. TyG and TyG/HDL-C were categorized into quartiles (Q1–Q4) based on their distributions in the overall sample, with Q1 as the reference group. In addition, TyG/HDL-C was standardized, and the OR per 1-standard-deviation increase was reported to facilitate interpretation and comparison.

#### 2.3.3 Dose–response assessment and nonlinearity testing

To characterize the dose–response relationship between continuous exposures and *H. pylori* positivity, restricted cubic splines (RCS) were incorporated into the logistic regression models. Knots were placed at the 5th, 35th, 65th, and 95th percentiles of the exposure distribution, with the median as the reference. Likelihood ratio tests were used to obtain *P*-values for the overall association and for nonlinearity.

#### 2.3.4 Bacterial activity–related load and metabolic indices among *H. pylori*–positive participants

Among *H. pylori*–positive participants (DOB ≥ 4‰), DOB was used as a proxy for bacterial activity–related load and was log-transformed [ln(DOB)] to reduce the impact of right-skewness. Linear regression models were fitted with TyG and TyG/HDL-C as outcomes using the same stepwise adjustment strategy (Models 1–3).

#### 2.3.5 Sensitivity analyses

To assess robustness, we performed the following sensitivity analyses, each using an extreme-value exclusion approach. (i) *H. pylori* status analyses (Model 3): (a) excluding participants with diabetes; (b) excluding participants with triglycerides above the 99th percentile (P99); and (c) excluding participants meeting both criteria. (ii) DOB-based analyses: repeating the regression among *H. pylori*–positive participants after excluding those with DOB above the 99th percentile (P99).

## 3 Results

### 3.1 Baseline characteristics by *Helicobacter pylori* status

A total of 705 participants were included, of whom 238 (33.8%) were *H. pylori*–positive. Compared with the *H. pylori*–negative group, the *H. pylori*–positive group had a higher proportion of males, higher alcohol use, and a higher prevalence of diabetes, while age was slightly lower and BMI was higher (all *P* < 0.05). Regarding metabolic indices, the *H. pylori*–positive group had higher triglycerides, TyG, and TyG/HDL-C, and lower HDL-C (all *P* < 0.01). No significant between-group differences were observed for smoking, hypertension, glucose, total cholesterol, or LDL-C (Table 1).

**TABLE 1:** Baseline characteristics by *H. pylori* status.

Variable	Overall (*n* = 705)	*H. pylori*-negative (*n* = 467)	*H. pylori*-positive (*n* = 238)	*P*-value	SMD
Male	348 (49.4%)	208 (44.5%)	140 (58.8%)	< 0.001	0.286
Smoking (yes)	149 (21.1%)	90 (19.3%)	59 (24.8%)	0.090	0.133
Alcohol drinking (yes)	100 (14.2%)	56 (12.0%)	44 (18.5%)	0.019	0.181
Hypertension (yes)	195 (27.7%)	124 (26.6%)	71 (29.8%)	0.357	0.073
Diabetes (yes)	68 (9.6%)	37 (7.9%)	31 (13.0%)	0.030	0.167
Age (years)	56.00 (48.00, 61.00)	57.00 (49.00, 61.00)	55.00 (45.00, 61.00)	0.024	0.184
BMI (kg/m^2^)	23.44 (21.09, 25.45)	23.01 (21.05, 24.99)	24.11 (21.40, 26.42)	< 0.001	0.306
DOB (‰)	2.07 (0.45, 9.33)	0.85 (0.20, 2.07)	20.14 (9.16, 36.43)	< 0.001	1.733
Glucose (mg/dL)	92.97 (86.67, 101.80)	92.61 (86.49, 100.54)	93.87 (86.89, 105.59)	0.105	0.237
Triglycerides (mg/dL)	112.49 (78.83, 166.52)	107.17 (77.95, 152.35)	123.56 (85.92, 192.43)	0.001	0.320
HDL-C (mg/dL)	45.63 (39.44, 54.52)	46.40 (39.44, 56.46)	43.70 (39.15, 51.82)	0.005	0.210
TyG	8.58 (8.18, 9.02)	8.51 (8.17, 8.89)	8.73 (8.24, 9.27)	< 0.001	0.324
TyG/HDL-C	0.19 (0.15, 0.22)	0.18 (0.15, 0.22)	0.20 (0.16, 0.23)	< 0.001	0.272

### 3.2 Associations of TyG and TyG/HDL-C with *H. pylori* positivity

**FIGURE 2:**
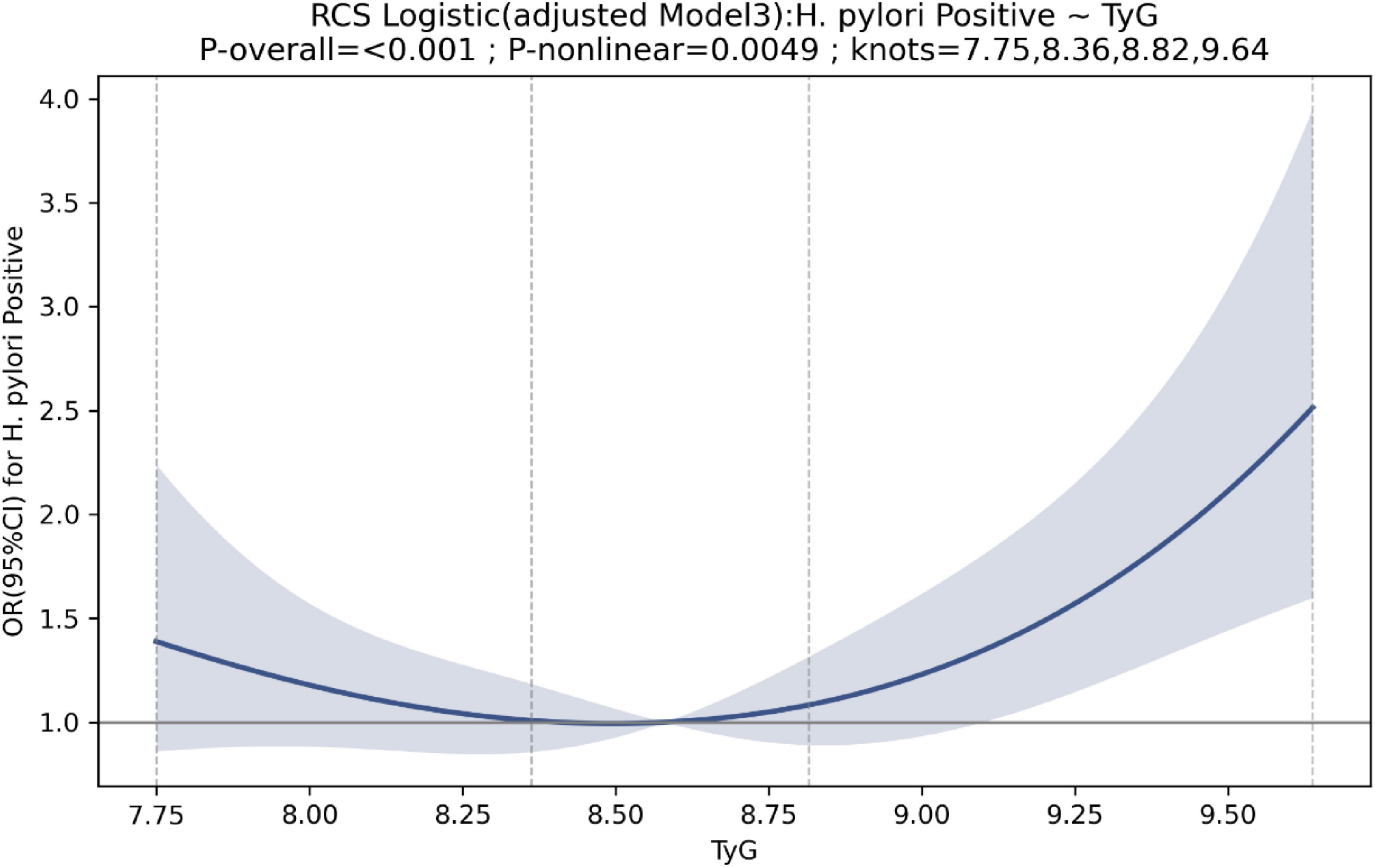
Restrictive cubic spline for the association between TyG and *Helicobacter pylori* positive.

In logistic regression models, TyG was independently associated with *H. pylori* positivity. In the unadjusted model, each 1-unit increase in TyG was associated with higher odds of *H. pylori* positivity (OR 1.735, 95% CI 1.34–2.25; *P* < 0.001). After adjustment for age and sex, the association remained significant (OR 1.66, 95% CI 1.27–2.17; *P* < 0.001). In the fully adjusted model (age, sex, BMI, smoking, alcohol use, hypertension, and diabetes), TyG remained independently associated with *H. pylori* positivity (OR 1.47, 95% CI 1.10–1.98; *P* = 0.010) (Table 2). In quartile analyses, Q4 (vs. Q1) showed higher odds of *H. pylori* positivity in the unadjusted and age-/sex-adjusted models (OR 1.923 and 1.79, respectively), but the association was attenuated and no longer significant in the fully adjusted model (OR 1.45; *P* = 0.133) (Table 2). Restricted cubic spline analyses indicated a nonlinear dose–response relationship between TyG and *H. pylori* positivity (P for overall < 0.001; P for nonlinearity = 0.0049). The curve showed an initial decrease followed by an increase, suggesting higher odds of *H. pylori* positivity at higher TyG levels (Figure 2).

**TABLE 2:** The association between TyG, TyG/HDL and the infection status of *Helicobacter pylori*.

Variables	Model 1		Model 2		Model 3	
	OR (95% CI)	*P*	OR (95% CI)	*P*	OR (95% CI)	*P*
TyG (continuous)	1.73 (1.34–2.25)	< 0.001	1.66 (1.27–2.17)	< 0.001	1.47 (1.10–1.98)	0.010
TyG (quartile)						
Q1	1.00 (reference)	–	1.00 (reference)	–	1.00 (reference)	–
Q2	0.70 (0.44–1.11)	0.133	0.70 (0.44–1.13)	0.144	0.67 (0.42–1.09)	0.108
Q3	1.01 (0.64–1.58)	0.971	0.99 (0.63–1.57)	0.971	0.86 (0.53–1.38)	0.525
Q4	1.93 (1.25–2.97)	0.003	1.79 (1.14–2.80)	0.011	1.45 (0.89–2.36)	0.133
TyG/HDL (continuous, per 1 SD)	1.31 (1.12–1.54)	< 0.001	1.24 (1.05–1.46)	0.011	1.16 (0.97–1.38)	0.102
TyG/HDL (quartile)						
Q1	1.00 (reference)	–	1.00 (reference)	–	1.00 (reference)	–
Q2	1.19 (0.75–1.90)	0.456	1.14 (0.71–1.82)	0.599	1.05 (0.65–1.70)	0.852
Q3	1.88 (1.20–2.95)	0.006	1.69 (1.06–2.68)	0.027	1.49 (0.92–2.41)	0.101
Q4	1.88 (1.20–2.95)	0.006	1.60 (1.00–2.57)	0.052	1.30 (0.79–2.15)	0.305

**FIGURE 3:**
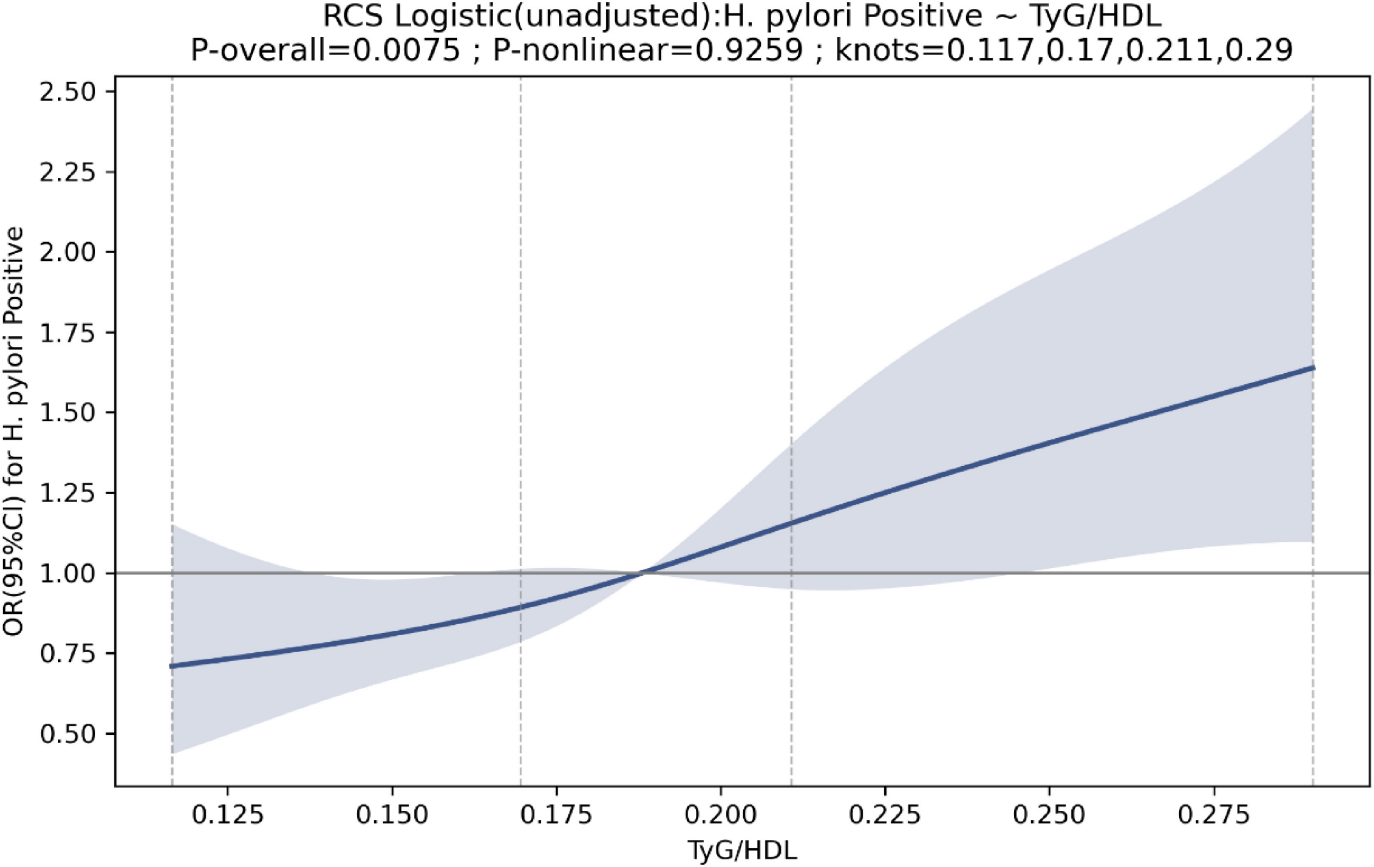
Restrictive cubic spline for the association between TyG/HDL and *Helicobacter pylori* positive.

Per 1-standard-deviation increase in TyG/HDL-C (SD = 0.0558), the odds of *H. pylori* positivity were higher in the unadjusted and partially adjusted models (OR 1.31 and 1.24, respectively), but the association was attenuated and not significant in the fully adjusted model (OR 1.16; *P* = 0.102). In quartile analyses, Q3 and Q4 (vs Q1) were associated with higher odds of *H. pylori* positivity in Models 1 and 2, whereas no significant differences were observed in the fully adjusted model (Table 2). Restricted cubic spline analyses did not suggest significant nonlinearity (Figure 3).

### 3.3 Sensitivity analyses

After excluding participants with diabetes (*n* = 637), TyG remained significantly associated with *H. pylori* positivity (per 1-SD increase: OR 1.267, 95% CI 1.047–1.534; *P* = 0.015), whereas the association for TyG/HDL-C was attenuated to borderline significance (OR 1.191, 95% CI 0.986–1.439; *P* = 0.070). After excluding participants with extreme triglyceride values (*n* = 697), the association for TyG remained significant (OR 1.245, 95% CI 1.033–1.500; *P* = 0.021), whereas TyG/HDL-C was not significant (OR 1.107, 95% CI 0.923–1.327; *P* = 0.275). After simultaneously excluding participants with diabetes and those with extreme triglyceride values (*n* = 631), the association for TyG remained robust (OR 1.267, 95% CI 1.040–1.544; *P* = 0.019), whereas TyG/HDL-C was not significant (OR 1.153, 95% CI 0.950–1.400; *P* = 0.149). Overall, the association between TyG and *H. pylori* positivity remained significant across sensitivity analyses, supporting the robustness of the primary finding. In contrast, the association for TyG/HDL-C was attenuated after full adjustment and was more sensitive to excluding participants with diabetes and extreme triglyceride values (Table 3).

**TABLE 3:** Sensitivity analyses for associations with *H. pylori* positivity (Model 3).

Variables	Main analysis (*n* = 705)		Excluding diabetes (*n* = 637)		Excluding TG > P99 (*n* = 697)		Excluding diabetes and TG > P99 (*n* = 631)	
	OR (95% CI)	*P*	OR (95% CI)	*P*	OR (95% CI)	*P*	OR (95% CI)	*P*
TyG (per +1)	1.472 (1.096, 1.978)	0.010	1.477 (1.078, 2.023)	0.015	1.434 (1.055, 1.951)	0.021	1.477 (1.067, 2.045)	0.019
TyG (per +1 SD)	1.265 (1.057, 1.513)	0.010	1.267 (1.047, 1.534)	0.015	1.245 (1.033, 1.500)	0.021	1.267 (1.040, 1.544)	0.019
TyG/HDL (per +1 SD)	1.158 (0.971, 1.381)	0.102	1.191 (0.986, 1.439)	0.070	1.107 (0.923, 1.327)	0.275	1.153 (0.950, 1.400)	0.149

Model 3 was adjusted for age, sex, BMI, smoking, drinking, hypertension, and diabetes (diabetes term omitted when excluded). TyG per 1 SD corresponds to SD = 0.6070; TyG/HDL per 1 SD corresponds to SD = 0.0558. TG P99 cutoff = 435.6778 mg/dL.

### 3.4 Additional subgroup and interaction analyses

Additional subgroup and interaction analyses were performed according to age ( < 60 vs. ≥ 60 years) and BMI ( < 24 vs. ≥ 24 kg/m^2^). The association between TyG and *H. pylori* positivity was stronger among participants with BMI ≥ 24 kg/m^2^ than among those with BMI < 24 kg/m^2^ (P for interaction = 0.009). A borderline interaction by age was also observed for TyG (P for interaction = 0.076), with the association appearing stronger in participants aged ≥ 60 years. For TyG/HDL-C, a significant interaction with age was identified (P for interaction = 0.025), whereas no significant interaction with BMI was observed (P for interaction = 0.646). Detailed subgroup-specific estimates are shown in Supplementary Table 2.

### 3.5 Association between DOB and TyG/TyG/HDL-C

Among *H. pylori*–positive participants, bacterial activity–related load [ln(DOB)] was not significantly associated with TyG or TyG/HDL-C in either unadjusted or fully adjusted models (all *P* > 0.05) (Table 4). After excluding two participants with DOB values above the 99th percentile (P99; *n* = 236), the results remained non-significant (Supplementary Table 1).

**TABLE 4:** The association between DOB-derived bacterial activity load and TYG, TYG/HDL.

Variables	Model 1		Model 2		Model 3	
	β (95% CI)	*P*	β (95% CI)	*P*	β (95% CI)	*P*
TyG	−0.0300 (−0.1382, 0.0782)	0.587	0.0105 (−0.0966, 0.1175)	0.848	0.0224 (−0.0754, 0.1201)	0.654
TyG/HDL	−0.0046 (−0.0134, 0.0042)	0.303	−0.0006 (−0.0094, 0.0082)	0.894	0.0006 (−0.0077, 0.0088)	0.896

## 4 Discussion

This study evaluated the associations of TyG and TyG/HDL-C with *Helicobacter pylori* infection status and breath-test–derived bacterial activity–related load. We found that TyG was independently associated with *H. pylori* positivity and exhibited a nonlinear dose–response pattern. TyG/HDL-C was associated with *H. pylori* positivity in unadjusted and partially adjusted models, but the association was attenuated and became non-significant after full adjustment for metabolic factors (e.g., BMI and diabetes), suggesting that this relationship may be more susceptible to confounding by metabolic comorbidities.

Among *H. pylori*–positive participants, DOB was not significantly associated with TyG or TyG/HDL-C. ^13^C-UBT–derived DOB values may partially reflect bacterial activity–related load; however, metabolic status may relate more to infection status than to the magnitude of bacterial activity–related load. Results were unchanged after excluding participants with extreme DOB values.

The finding that TyG was associated with *H. pylori* positivity, whereas ln(DOB) was not associated with TyG or TyG/HDL-C among infected participants, may suggest that infection status and DOB-derived bacterial activity load reflect different biological dimensions. The former may be more closely related to host susceptibility, chronic inflammatory responses, and the metabolic phenotype associated with established infection, whereas the latter may more directly reflect urease activity–related signals and be influenced by bacterial characteristics, the gastric microenvironment, and testing conditions (6–8). Therefore, DOB levels may not necessarily show a linear relationship with TyG or TyG/HDL-C.

In sensitivity analyses, the association between TyG and *H. pylori* positivity remained significant, supporting the robustness of the findings. Overall, TyG may better capture the metabolic phenotype associated with *H. pylori* infection status.

It should be noted that TyG and TyG/HDL-C are not disease-specific markers and may be influenced by multiple host metabolic factors, underlying diseases, and comorbid conditions (9, 10). Prior studies have shown that these indices are closely related to glucose–lipid metabolic abnormalities, cardiometabolic risk, and chronic metabolic diseases. Therefore, the associations observed in the present study are unlikely to represent a direct effect of *H. pylori* on these indices, but are more likely mediated by secondary host responses to infection. Persistent *H. pylori* infection may induce gastric mucosal inflammation and contribute to chronic low-grade systemic inflammation (11, 12); inflammatory mediators and oxidative stress may further disrupt glucose and lipid metabolism and promote insulin resistance. In addition, alterations in gastrointestinal hormone secretion, lipid remodeling, and gut microbiota may also be involved (13). Notably, the association between TyG and *H. pylori* positivity remained stable across multiple sensitivity analyses, suggesting that this relationship is not entirely driven by diabetes or extreme triglyceride values.

As a simple surrogate marker of insulin resistance, TyG has been widely used for cardiometabolic risk assessment and stratification (4). Our findings that TyG is independently associated with *H. pylori* positivity are consistent with recent studies (14, 15). Persistent *H. pylori* infection can induce gastric mucosal inflammation and may contribute to a chronic systemic inflammatory milieu (11). Inflammatory mediators can disrupt glucose and lipid metabolism (12), thereby promoting insulin resistance. Chronic inflammation may also impair lipoprotein function and limit triglyceride clearance (13). In addition, chronic infection may alter gastrointestinal hormone secretion and fat distribution. *H. pylori* may influence the gut microbiota, and some studies have reported improvements in lipid profiles and remodeling of the gut microbiota after eradication therapy (16).

Substantial prior evidence has linked *H. pylori* infection to metabolic abnormalities and insulin resistance (5, 17). Several studies have reported a higher risk of metabolic syndrome or insulin resistance among *H. pylori*–infected individuals (5), whereas others have found no significant association, potentially due to differences in study populations, diagnostic methods, and confounder adjustment (18–20). Across different regions, our findings are broadly consistent with some international studies. Population-based analyses from the United States, such as NHANES, have also shown that higher TyG is associated with an increased likelihood of *H. pylori* infection even after adjustment for demographic and metabolic factors (14, 15). On the other hand, studies from other regions have not consistently observed significant associations between *H. pylori* infection and metabolic syndrome or insulin resistance, particularly in populations with specific underlying diseases, where adjustment for confounders may attenuate or eliminate the association (18, 19). These discrepancies may reflect differences in study populations, diagnostic methods for *H. pylori*, baseline metabolic risk, and the degree of confounder control. Importantly, our study was conducted in a single-center hospitalized Chinese population, in which clinical heterogeneity and metabolic comorbidity burden may be greater than in community-based cohorts; thus, our findings provide complementary real-world evidence from an East Asian inpatient setting. Consistent with several cross-sectional studies, we observed that higher TyG was associated with greater odds of *H. pylori* infection even after multivariable adjustment (14, 15). However, the RCS curve for TyG and *H. pylori* positivity was U-shaped, suggesting that the relationship in hospitalized patients may not be strictly monotonic or linear (21). This pattern may differ from that observed in other populations and could reflect clinical heterogeneity and residual confounding (18). Additional subgroup analyses further suggested that the TyG–*H. pylori* association was more pronounced among participants with higher BMI, whereas the TyG/HDL-C association appeared to vary by age. These findings support the possibility that host metabolic background and age-related heterogeneity may modify the observed associations.

Regarding TyG/HDL-C, some studies have reported an association with *H. pylori* infection that becomes non-significant after confounder adjustment (22), consistent with the notion that different insulin-resistance surrogates vary in their sensitivity to confounding (23).

With respect to bacterial activity–related load, prior studies have stratified ^13^C-UBT DOB values to examine associations with glucose–lipid metabolic abnormalities, suggesting a possible relationship, although evidence remains limited (6). DOB reflects a urease activity–related signal and does not directly quantify bacterial burden (7). Therefore, DOB levels may not relate linearly to TyG or TyG/HDL-C (8), consistent with our findings.

This study has several limitations. Its single-center, retrospective cross-sectional design limits causal inference and generalizability. In addition, information on lipid-lowering medications was unavailable, which may have introduced unmeasured confounding. Similarly, information on glucose-lowering medications was not systematically available, which may also have contributed to residual confounding in the analyses of TyG and TyG/HDL-C. No follow-up data after *H. pylori* eradication therapy were available; therefore, we could not determine whether TyG or TyG/HDL-C changed after bacterial clearance. Although baseline differences were addressed by multivariable adjustment, SMD assessment, sensitivity analyses, and additional subgroup/interaction analyses, residual confounding cannot be completely excluded. Future prospective, multicenter studies incorporating medication data, inflammatory markers, and repeated measurements are warranted to confirm these findings and clarify underlying mechanisms.

## 5 Conclusion

TyG is independently associated with *H. pylori* positivity and exhibits a nonlinear dose–response relationship. In contrast, the association between TyG/HDL-C and *H. pylori* positivity is attenuated and becomes non-significant after full adjustment. DOB is not significantly associated with TyG or TyG/HDL-C among *H. pylori*–positive participants.

## Data Availability

The raw data supporting the conclusions of this article will be made available by the authors, without undue reservation.
