# Supplementary material for: Association of TyG and TyG/HDL with Helicobacter pylori infection status and urea breath test load: a cross-sectional study
Source: Front Med (Lausanne). 2026 May 8;13:1824476. doi: 10.3389/fmed.2026.1824476 (PMC13195007; doi:10.3389/fmed.2026.1824476)
Supplement: Supplementary file 1 [file Table_1.docx]

**Table S1 Sensitivity analysis: Associations of lnDOB with TyG and TyG/HDL after excluding DOB>P99**

| **Outcome** | **Model 1** | | **Model 2** | | **Model 3** | |
| --- | --- | --- | --- | --- | --- | --- |
|  | **β (95% CI)** | **P** | **β (95% CI)** | **P** | **β (95% CI)** | **P** |
| TyG | -0.0098 (-0.1174, 0.0978) | 0.858 | 0.0283 (-0.0786, 0.1353) | 0.604 | 0.0327 (-0.0671, 0.1324) | 0.521 |
| TyG/HDL | -0.0038 (-0.0127, 0.0050) | 0.397 | 0.0001 (-0.0088, 0.0090) | 0.984 | 0.0006 (-0.0079, 0.0092) | 0.883 |

Hp-positive definition: DOB≥4‰. P99 from SPSS: 82.2461. Excluded DOB>P99: 2; remaining n=236. Exposure: lnDOB=ln(DOB). Model 1: unadjusted; Model 2: adjusted for age and sex; Model 3: further adjusted for BMI, smoking, drinking, hypertension, and diabetes. Robust standard errors (HC3).
