# Supplementary material for: Association of TyG and TyG/HDL with Helicobacter pylori infection status and urea breath test load: a cross-sectional study
Source: Front Med (Lausanne). 2026 May 8;13:1824476. doi: 10.3389/fmed.2026.1824476 (PMC13195007; doi:10.3389/fmed.2026.1824476)
Supplement: Supplementary file 2 [file Table_2.docx]

**Table S2. Subgroup and interaction analyses for associations of TyG and TyG/HDL-C with H. pylori positivity**

| **Variables** | **Age group** | | | | | **BMI group** | | | | |
| --- | --- | --- | --- | --- | --- | --- | --- | --- | --- | --- |
|  | **<60 OR (95% CI)** | **P** | **≥60 OR (95% CI)** | **P** | **P for interaction** | **<24 OR (95% CI)** | **P** | **≥24 OR (95% CI)** | **P** | **P for interaction** |
| TyG | 1.185 (0.823, 1.706) | 0.361 | 2.160 (1.248, 3.738) | 0.006 | 0.076 | 1.062 (0.714, 1.580) | 0.767 | 2.356 (1.497, 3.708) | <0.001 | 0.009 |
| TyG/HDL-C (per 1 SD) | 0.972 (0.772, 1.222) | 0.805 | 1.499 (1.113, 2.019) | 0.008 | 0.025 | 1.134 (0.891, 1.443) | 0.307 | 1.198 (0.926, 1.550) | 0.169 | 0.646 |

Model 3 was used for all subgroup analyses. Age strata were defined as <60 and ≥60 years. BMI strata were defined as <24 and ≥24 kg/m². TyG/HDL-C was analyzed per 1-SD increase. P for interaction was derived from the cross-product term in the fully adjusted logistic regression model.
